# Improvement of ethanol production by ethanol-tolerant *Saccharomyces cerevisiae* UVNR56

**DOI:** 10.1186/2193-1801-2-583

**Published:** 2013-10-31

**Authors:** Sutticha Na-Ranong Thammasittirong, Thanawan Thirasaktana, Anon Thammasittirong, Malee Srisodsuk

**Affiliations:** Microbial Biotechnology Unit, Department of Microbiology, Faculty of Liberal Arts and Science, Kasetsart University Kamphaeng Saen Campus, Nakorn Pathom, 73140 Thailand; Department of Science, Faculty of Liberal Arts and Science, Kasetsart University Kamphaeng Saen Campus, Nakorn Pathom, 73140 Thailand

**Keywords:** Ethanol production, Ethanol tolerance, Molasses, Mutagenesis, *Saccharomyces cerevisiae*

## Abstract

Ethanol tolerance is one of the important characteristics of ethanol-producing yeast. This study focused on the improvement of ethanol tolerance of *Saccharomyces cerevisiae* NR1 for enhancing ethanol production by random UV-C mutagenesis. One ethanol-tolerant mutant, UVNR56, displayed a significantly improved ethanol tolerance in the presence of 15% (v/v) ethanol and showed a considerably higher viability during ethanol fermentation from sugarcane molasses and sugarcane molasses with initial ethanol supplementation. A maximum ethanol concentration produced from molasses medium at 37°C by UVNR56 was 10.3% (v/v), productivity of 1.7 g/l/h and a theoretical yield of 98.7%, while the corresponding values for the wild-type were 8.6% (v/v), 1.4 g/l/h and 83.3%, respectively. In addition, during molasses fermentation under initial supplementation of 5% (v/v) ethanol, the maximum ethanol concentration and productivity of UVNR56 was 25.7% and 42.9% higher than the wild-type, respectively.

## Background

Ethanol is an attractive renewable biofuel. Increasing the availability of this alternative energy source requires ethanologenic yeasts that can produce ethanol more efficiently. During ethanol fermentation, yeasts are exposed to various stresses, including increased ethanol, toxic by-product inhibition, high temperature and osmotic pressure from high concentrations of substrate sugar. Among the factors, ethanol is considered to be the major stress responsible for decreased ethanol production and stuck fermentation (Gibson et al. 2007). At concentrations in excess of 8% (v/v) ethanol cause the phospholipid of the lipid bilayer of cell membranes and organelles, such as the inner membrane of mitochondria, to become hyperpolarized thereby increasing membrane fluidity and consequentially decreasing membrane integrity (Lloyd et al. 1993; Ly et al. 2002; Mishra and Prasad 1989). When the cell membrane becomes more permeable to small molecules and ions, the perturbation of cell homeostasis impacts on several cellular metabolic pathways (Walker 1998). High ethanol production capability of ethanologenic yeasts under the presence of high ethanol is one of the most important factors for ethanol production. The development of such strains is of great economic value to industries involved in fermenting, distilling and refining ethanol. In the ethanol industry, ethanol production is usually among 10-14% (v/v) and the theoretical yield has to be as high as 90-93% of the fermentation efficiency for the conversion of glucose into ethanol (Bai et al. 2008). Consequently, several studies to date have focused interest on ethanol tolerance of ethanol-producing yeasts based on the presumption that ethanol-tolerant yeast strains would have enhanced ethanol productivities and yields (Fiedurek et al. 2011: Shi et al. 2009; Thammasittirong et al. 2012).

In the present study, an existing UV-C treated library of *Saccharomyces cerevisiae* was screened for selection mutants with high sugarcane molasses fermentation ability. The mutants were further treated with UV-C radiation to increase ethanol production by improving ethanol tolerance. The ethanol tolerance and fermentation characteristics of the ethanol-tolerant mutants in molasses medium were examined in comparison to the wild-type. In addition, ethanol fermentation from molasses medium in the presence of stressful levels of ethanol was also investigated.

## Results and discussion

### UV-C mutagenesis and ethanol tolerance of the mutants

**Figure 1:**
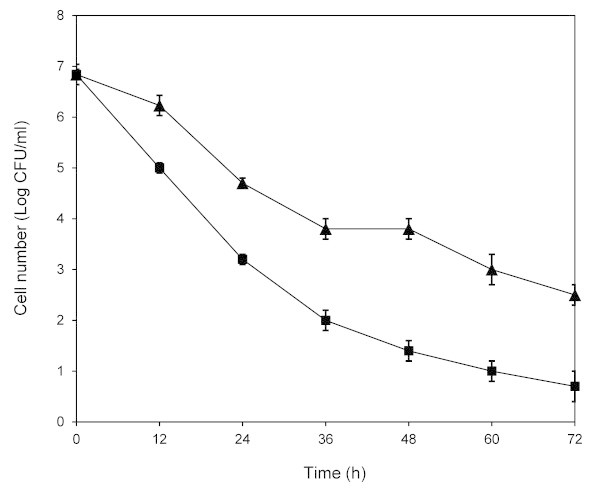
**Comparison of viable cells during cultivation in YPD medium containing 15% (v/v) ethanol of UVNR56 (** ***filled triangle*** **) and the wild-type (** ***filled square*** **).** All experiments were performed at 30°C with 100 rpm. Cell samples were taken and spreading serially diluted samples on YPD agar. Colonies were counted after 48 h incubation at 30°C. Data represent the mean ± standard deviation from three independent experiments.

The results of the shake-flask fermentation of the mutants from our previous UV-C mutant library were that five of 16 colonies fermented in sugarcane molasses medium produced 3-10% more ethanol than the wild-type (NR1) (data not shown). In order to increase ethanol production from sugarcane molasses by improving ethanol tolerance, the selected five mutants were pooled and then subjected to further UV-C mutagenesis. The colonies that grew in molasses medium at the higher ethanol concentration on 12% (v/v) ethanol gradient plate, relative to the wild-type, were selected and analyzed for their ethanol tolerance ability according to the effect of ethanol on yeast viability. One ethanol-tolerant mutant, UVNR56, showed higher numbers of viable cells comparing to the wild-type in the presence of 15% (v/v) ethanol in YPD medium (Figure 1). YPD medium had been used as a growth medium in order to avoid the complexity and adaptation to environmental stresses of sugarcane molasses and to ensure that viability are directly connected with any ethanol effect. The high cell count indicated that UVNR56 possessed improved tolerance against ethanol. This ethanol-tolerant mutant strain retained its tolerant phenotype, even after twenty cycles of growth.

### Ethanol production from sugarcane molasses

**Figure 2:**
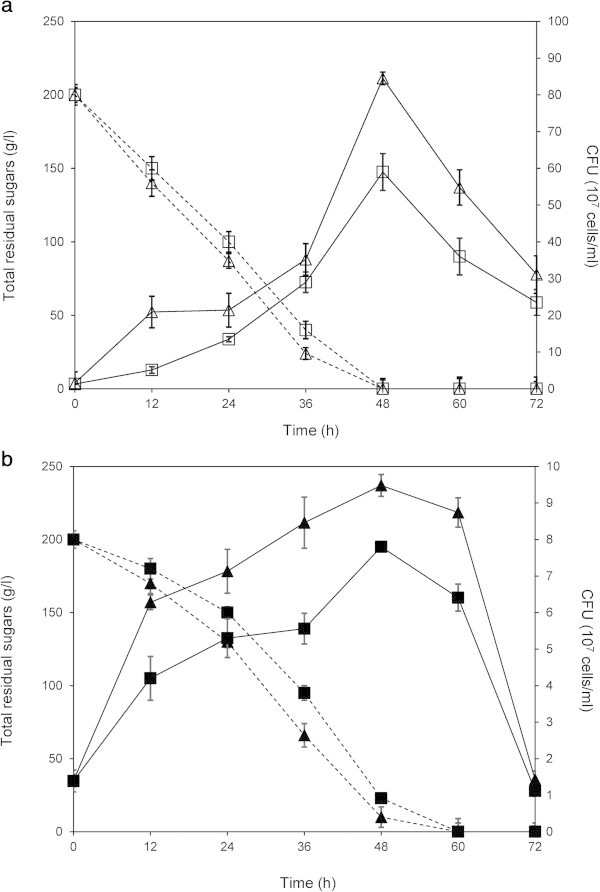
**Changes in measured parameters of viable cell count (** ***solid lines*** **) and residual sugars (** ***dashed lines*** **) a during molasses fermentation of UVNR56 (** ***unfilled triangle*** **) and the wild-type (** ***unfilled square*** **) and b during molasses fermentation with 5% (v/v) initial ethanol supplementation of UVNR56 (** ***filled triangle*** **) and the wild-type (** ***filled square*** **).** All experiments were performed at 37°C with 100 rpm. Data represent the mean ± standard deviation from three independent experiments.

In Thailand, sugarcane molasses is the basis of the major raw material for the fuel ethanol industry. Therefore, the viability, growth and fermentation properties in molasses medium of the ethanol-tolerant mutant, UVNR56, along with the wild-type were investigated further. The results revealed that UVNR56 displayed higher numbers of viable cells than the wild-type during molasses fermentation (Figure 2a). The highest value of a viable cell count of UVNR56 was found at 48 h of fermentation, which was 30% higher than the wild-type. Biomass and the ability of UVNR56 to utilize sugar in sugarcane molasses were slightly higher than those of the wild-type (Table 1 and Figure 2a). The products of the Browning reaction, furfural and 5-hydroxymethylfurfural, are known to be inhibitors of growth and ethanol fermentation by ethanologenic yeasts (Modig et al. 2002; Sarvari Horvath et al. 2003). With regard to viability, growth rate and sugar consumption in molasses of UVNR56, these results suggested that UVNR56 not only exhibited remarkably enhanced ethanol tolerance, but may also showed improved resistance to environmental stress in sugarcane molasses. Yeast cells with high viability and rapid growth are the important factors to increase ethanol production rate and reduce the cost of seed culture.

**Table 1:** **Ethanol production by the ethanol**-**tolerant mutant and the wild**-**type in molasses medium and molasses medium with 5**% (**v**/**v**) **initial ethanol supplementation at 37**°**C for 72 h**

Strain	Molasses medium				Molasses medium with added ethanol ^**^			
	Ethanol ^1^ % (v/v)	Productivity (g/l/h)	Theoretical ^*^ yield (%)	Biomass (g/l)	Ethanol ^1^ % (v/v)	Productivity (g/l/h)	Theoretical ^*^ yield (%)	Biomass (g/l)
NR1	8.6 ± 0.2^a^ (48 h)	1.4 ± 0.04^a^	83.3^a^	11.1 ± 0.3^a^	7.5 ± 0.2^a^ (72 h)	0.8 ± 0.04^a^	72.6^a^	8.9 ± 0.1^a^
UVNR56	10.3 ± 0.1^b^ (48 h)	1.7 ± 0.02^b^	98.7^b^	12.9 ± 0.2^b^	10.1 ± 0.1^b^ (60 h)	1.4 ± 0.02	97.9^b^	10.8 ± 0.2^b^

Different letters (a and b) in each column indicate significant differences between the yeast strains (*p* < 0.05).

^1^The time points indicate the maximum ethanol concentrations produced by the yeast strains.

^*^The following theoretical values have been used for calculation (g ethanol/ g sugar): sucrose, 0.538; maltose, 0.538; glucose, 0.511; fructose, 0.511. The values of composition of fermentable sugars in molasses (78% (w/v)) have been used for calculation (% w/w): sucrose, 33.5; fructose, 12.6; glucose, 10.2; maltose, 0.03. Theoretical yield of undiluted molasses = 0.296 g ethanol/ g molasses.

^**^Ethanol production = final ethanol - ethanol added to the medium.

UVNR56 displayed a higher ethanol concentration (10.3% (v/v)), productivity (1.7 g/l/h) and theoretical yield (98.7%) compared to the wild-type (Table 1). The results suggested that the enhancement of ethanol production of UVNR56 was mainly due to the improvement of ethanol tolerance, growth and sugar consumption. In addition, the ability to resist stress conditions in molasses may also be improved in UVNR56, and this could be the one of factors improving cell viability and ethanol production of this strain. Tolerance of ethanol and toxic compounds is well-known as a complex phenotype controlled by multiple genes (Ding et al. 2012; Hu et al. 2007; Teixeira et al. 2009). A deeper understanding of the mechanism of tolerance to ethanol and other environmental stresses, at a systems level, will facilitate the development of more tolerant and fermentation-efficient strains (Li et al. 2012).

### Ethanol production from sugarcane molasses with initial ethanol supplementation

As yeast cells are exposed to external ethanol during ethanol fermentation, we therefore decided to supplement molasses medium with 5% (v/v) ethanol. Under these conditions, yeast cells were exposed to high concentrations of ethanol, both exogenous and secreted into the medium. Viable cell counts of UVNR56 were significantly higher than the wild-type, but its viability was lower compared to the no added ethanol conditions (Figure 2b). Several studies have shown that an improvement in ethanol tolerance leads to an increase in both ethanol productivity and yield (Basso et al. 2008; Fiedurek et al. 2011; Hou 2009). As expected, we found a correlation between cell viability and fermentation capability. UVNR56 could effectively produce ethanol in the presence of stressful ethanol concentrations. The highest ethanol production of UVNR56 was 10.1% (v/v), which was 25.7% higher than that of the wild-type. Ethanol production rate of UVNR56 was also considerably faster than the wild-type (Table 1). UVNR56 showed productivity of 1.4 g/l/h, while the wild-type showed productivity of 0.8 g/l/h.

Supplementation of ethanol also resulted in slow sugar consumption rate of UVNR56 and the wild-type (Figure 2b). The amount of utilized sugar was almost completely consumed by both yeast strains within 60 h, while in conditions without exogenous ethanol, sugars were completely consumed within 48 h (Figure 2a and b). Biomass also decreased when cells were grown in the presence of added ethanol (Table 1).

The commercial strains of *S*. *cerevisiae* usually grow and produce ethanol between 30°C and 32°C, their ethanol productions and ethanol theoretical yields are usually among 10-14% (v/v) and 90-93%, respectively (Bai et al. 2008). Therefore, the high ethanol production (10.3% (v/v)) and the high ethanol yield (98%) of UVNR56 from ethanol fermentation at 37°C demonstrated the successfully generation of a genetically stable strain, capable of high ethanol tolerance and high ethanol production from sugarcane molasses by UV-C mutagenesis. UVNR56 is an excellent strain that is promising candidate for large-scale ethanol production from sugarcane molasses. Further examination of this strain under scale-up conditions and ethanol production at high temperature are planned for further study.

## Methods

### Yeast strain

*Saccharomyces cerevisiae* NR1 was isolated from soil sample and identified by nucleotide sequence analysis of the D1/D2 domain of 26S rDNA.

### Preparation of gradient plate

Gradient plate was prepared by a slight modification to the method of Szybalski (Szybalski and Bryson 1952). The lower layer consists of 10 ml of molasses medium (30% (w/v) soluble solid of sugarcane molasses, 0.05% (w/v) (NH_4_)_2_SO_4_, 0.05% (w/v) KH_2_PO_4_, 0.05% (w/v) MgSO_4_⋅7H_2_O). The petri plate was raised sufficiently to cover the entire bottom. After the medium solidified, the plate was placed in the normal horizontal and another 10 ml of molasses medium containing 12% (v/v) ethanol was added.

### UV-C mutagenesis

One ml of yeast cells suspension (approximately 1×10^6^ cells/ml) was spread on a gradient plate prepared as described above. The plates were exposed to UV-C rays (234 nm) at a distance of 30 cm with interval of 5, 10, 15 and 20 s and incubated at 30°C for 72 h.

### Determination of ethanol tolerance

Ethanol tolerance determination was carried out in YPD medium (1% (w/v) yeast extract, 2% (w/v) peptone, 2% (w/v) D-glucose) containing 15% (v/v) ethanol. Yeasts were inoculated in 100 ml YPD medium to achieve an initial cell density of 1 × 10^7^ cells/ml. The culture was grown with shaking at 100 rpm at 30°C. Cell samples were taken and spreading serially diluted samples on YPD agar. Colonies were counted after 48 h incubation at 30°C.

### Ethanol fermentation

Ethanol fermentations were performed at 37°C with cells initially adjusted to cell density of 1 × 10^5^ cells/ml in 100 ml molasses medium consisting of 28% (w/v) soluble solid without or with initial 5% (v/v) ethanol supplementation. Fermentation samples were taken every 12 h for determining viable cell count, ethanol concentration, reducing sugar in the culture and biomass of yeast.

### Analytical methods

The ethanol and sugar concentrations were analyzed by high-performance liquid chromatography (HPLC) using a sugar pak I column at 85°C and a refractive index detector. The mobile phase was deionized water at a flow rate of 1 ml/min. Biomass concentration was measured by gravimetric method after drying to constant weight.
